# Cross-Linking and Functional Analyses for Dimerization of a Cysteine Mutant of Glycine Transporter 1

**DOI:** 10.3390/ijms232416157

**Published:** 2022-12-18

**Authors:** Jingru Wang, Hanhe Liu, Yuan-Wei Zhang

**Affiliations:** School of Life Sciences, Guangzhou University, Guangzhou 510006, China

**Keywords:** glycine transporter, dimerization, cross linking, transport function, transport mechanism

## Abstract

Glycine transporter 1 (GlyT1) is responsible for the reuptake of glycine, which regulates glutamate signaling as a co-agonist with N-methyl-D-aspartic acid (NMDA) receptors in the excitatory synapse and has been proposed to be a potential target in the development of therapies for a broad range of disorders of the central nervous system. Despite significant progress in characterizing structure and transport mechanism of the transporter, the regulation of transport function through oligomerization remains to be understood. In the present work, association of two forms of GlyT1 into dimers and higher order oligomers was detected by coimmunoprecipitation. To investigate functional properties of dimers of a GlyT1 cysteine mutant L288C, we performed oxidative cross-linking of the positioned cysteine residues in extracellular loop 3 (EL3) near the extracellular end of TM6. By analyzing the effect of copper phenanthroline (CuP)-induced dimerization on transport function, cross-linking of L288C was found to inhibit transport activity. In addition, an intramolecular ion pair Lys286-Glu289 was revealed to be critical for stabilizing EL3 in a conformation that modulates CuP-induced dimerization and transport function of the GlyT1 L288C mutant. Furthermore, the influence of transporter conformation on GlyT1 L288C dimerization was investigated. The substrate glycine, in the presence of both Na^+^ and Cl^−^, significantly reduced oxidative cross-linking, suggesting a large-scale rotation of the bundle domain during substrate transport impairs interfacial interactions between L288C protomers. The present study provides new insights into structural and functional elements regulating GlyT1 transport activity through its dimerization or oligomerization.

## 1. Introduction

Glycine transporters (GlyTs) are responsible for the reuptake of glycine, an essential neurotransmitter that functions at both the inhibitory and excitatory synapses in the central nervous system. At inhibitory synapses, glycine activates postsynaptic glycine receptors to cause hyperpolarization [1,2], whereas it acts as a co-agonist with glutamate at N-methyl-D-aspartic acid (NMDA) receptors in the excitatory synapses [3]. These synaptic actions of glycine are regulated by two subtypes of GlyTs, glycine transporter 1 (GlyT1) and glycine transporter 2 (GlyT2), which share ~50% sequence identity but differ in tissue distribution [4]. GlyT1 is of particular interest in neuropharmacology because it participates in the regulation of NMDA receptor activity through control of glycine concentrations at the excitatory synapses [5,6]. Consequently, GlyT1 has been proposed as a potential target for the treatment of several disorders, including schizophrenia [7,8,9], drug addiction [10], and neuropathic pain [11,12,13].

GlyTs belong to a large family of neurotransmitter sodium symporters (NSS, SLC6), which also includes transporters for serotonin (SERT), dopamine (DAT), norepinephrine (NET), and γ-aminobutyric acid (GAT). The NSS transporters utilize transmembrane ion gradients of Na^+^ and Cl^−^ to drive substrates across the membrane by an alternating access mechanism in which the central binding site is alternately exposed to extracellular or cytoplasmic medium for substrate binding and release, with conformational changes opening and closing the substrate permeation pathways [14,15,16,17]. X-ray structures of the bacterial NSS amino acid transporter LeuT in several conformational states provide insights into these conformational changes in the transport cycle [18,19,20]. Furthermore, the recently resolved structures of DAT, SERT, and GlyT1 reveal these transport proteins in similar conformations, indicating that they share a common transport mechanism [21,22,23,24,25,26].

Despite significant progress in characterizing structures and transport mechanisms of NSS transporters, many aspects of the regulation of transport function remain to be understood. One challenge is to understand the mechanism of transporter oligomerization and its significance in regulating transport function. Earlier biochemical studies have confirmed the presence of dimers and higher order oligomers of the NSS transporters in the plasma membrane, however, stoichiometry and structural arrangements of the oligomeric quaternary structures of the NSS transporters remained to be determined [27,28,29,30,31,32,33,34,35,36,37].

LeuT has been crystallized as a dimer with TM9 and TM12 in the interface [18]. By comparison, the same dimer formation has not been observed in the eukaryotic NSS transporters, DAT, SERT, and GlyT1, possibly due to a pronounced kink in the middle of TM12 that was found in high-resolution structures of these proteins [21,22,23,24,25,26], suggesting that they form dimers by a mechanism different from that of LeuT. Based on cross-linking experiments with DAT, in which an endogenous Cys306 residue was identified in the dimeric interface [38], both GlyT1 and GlyT2 were also detected as dimers in the plasma membrane by mutating and cross-linking the equivalent position [39]. The corresponding residues in GlyT1 and GlyT2, thus, were proposed to contribute to the dimeric interface [39]. However, this previous study did not address either functional properties of cross linker-induced dimerization of GlyT1 and GlyT2 or the molecular factors regulating their cross-linking.

In this present work, we have constructed two epitope-tagged forms of GlyT1, which allow us to directly measure association of detergent-solubilized GlyT1 by coimmunoprecipitation. In addition, by oxidative cross-linking, we investigated the effect of dimerization of cysteine mutant L288C of GlyT1 on its transport properties. Furthermore, we examined the influence of an intramolecular ion pair on L288C dimerization and transport activity. Finally, we also investigated the influence of Zn^2+^ and GlyT1 conformations on cross-linking of L288C. Our results support the proposal that oxidation-induced dimerization of GlyT1 L288C has a profound effect on its transport function.

## 2. Results

### 2.1. Association of FLAG-GlyT1 with GlyT1-HA

**Figure 1:**
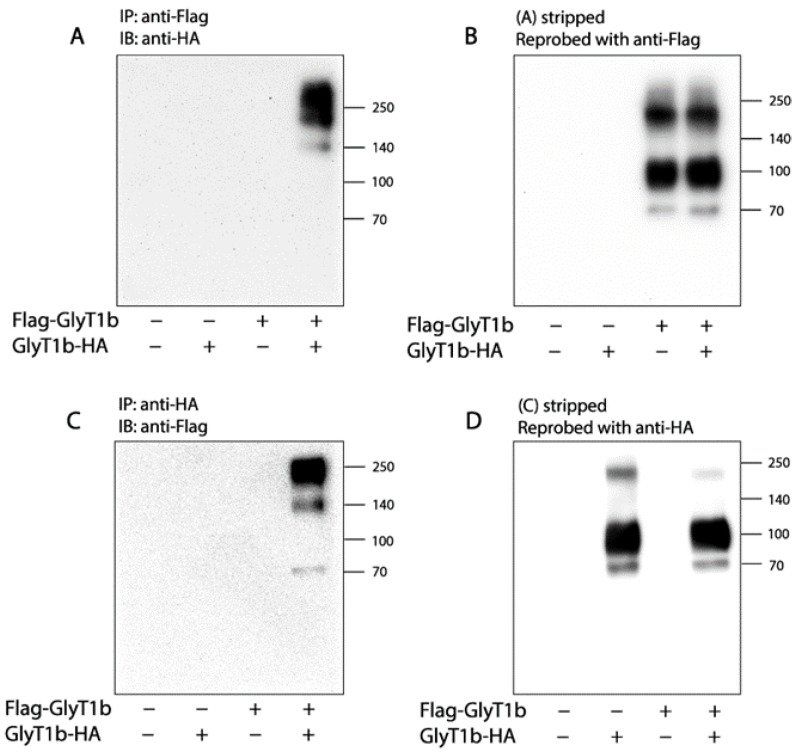
Association of two epitope-tagged GlyT1 forms. (**A**,**B**) Co-immunoprecipitation of FLAG-GlyT1 with GlyT1-HA. FLAG-tagged GlyT1 in total lysates of the cells co-expressing FLAG-GlyT1 with GlyT1-HA was pulled down by anti-FLAG agarose beads and subjected to separation by SDS-PAGE. The HA-tagged GlyT1 associated with FLAG-GlyT1 was then detected by immunoblot analysis with anti-HA antibody (**A**). After stripping, the immunoprecipitated FLAG-tagged GlyT1 was detected by re-probing the membrane with anti-FLAG antibody (**B**). (**C**,**D**) Co-immunoprecipitation of GlyT1-HA with FLAG-GlyT1. The HA-tagged GlyT1 was pulled down from cell lysates using anti-HA agarose beads and separated by SDS-PAGE. The FLAG-GlyT1 associated with GlyT1-HA was detected by immunoblot analysis with anti-FLAG antibody (**C**). Immunoprecipitation of the HA tagged GlyT1 was confirmed by anti-HA antibody (**D**). Graphs show representative blots, and experiments were performed more than three times with similar results.

To test the ability of two epitope-tagged forms of GlyT1 to associate in detergent solution as a measure of GlyT1 dimerization or oligomerization, we constructed expression plasmids for GlyT1b with a FLAG-tag at N-terminus or a HA-tag at C-terminus. HEK293 cells co-expressing both GlyT1 forms with a transfection mixture of two plasmids at a 1:1 ratio were solubilized with an RIPA buffer, and FLAG-GlyT1 or GlyT1-HA was pulled down with anti-FLAG M2 or anti-HA agarose beads and analyzed for its association with the other epitope-tagged GlyT1 form, respectively. As shown in Figure 1A, no GlyT1-HA signals in anti-FLAG immunoprecipitates were detected by immunoblot analysis when the cells only expressed either FLAG-GlyT1 or GlyT1-HA. In contrast, in the cells co-expressing both FLAG-GlyT1 and GlyT1-HA, prominent immunoreactive HA bands at ~140 kDa (a dimer of non-glycosylated form), ~190 kDa (a dimer of fully glycosylated form), and more than 250 kDa (higher order oligomers) were detected in anti-FLAG immunoprecipitates, indicating that the two forms of GlyT1 remained associated in the RIPA buffer containing 1% Triton X-100. The blot was then stripped and reprobed with an anti-FLAG antibody (Figure 1B). FLAG-GlyT1 bands at ~70 kDa (a monomer of non-glycosylated form), ~95 kDa (a monomer of fully glycosylated form), and ~190 kDa (a dimer of fully glycosylated form) were detected only in immunoprecipitates from the cells expressing FLAG-GlyT1 (right two lanes), suggesting the FLAG-GlyT1 and GlyT1-HA specifically associated with each other.

Similarly, we performed immunoblot analysis to detect association of the two GlyT1 forms using anti-HA agarose beads to pull down GlyT1. As shown in Figure 1C,D, FLAG-GlyT1 bands were detected in anti-HA immunoprecipitates only from the cells co-expressing GlyT1-HA with FLAG-GlyT1. By comparison, in neither case using cells expressing FLAG-GlyT1 or GlyT1-HA alone was a FLAG-GlyT1 band detected in the immunoblot. The observation supports the specificity of association of the two GlyT1 forms.

We noticed the GlyT1-HA signals detected in anti-FLAG immunoprecipitates with a molecular mass pattern different from that of FLAG-GlyT1 detected after the blot was stripped and reprobed with an anti-FLAG antibody (rightmost lanes in Figure 1A,B). Similar results were also observed when FLAG-GlyT1 and GlyT1-HA were detected in anti-HA immunoprecipitates (rightmost lanes in Figure 1C,D). To see if those GlyT1 forms in anti-FLAG or anti-HA immunoprecipitates exist in total lysates from the cells expressing FLAG-GlyT1, GlyT1-HA or both, we performed immunoblot analysis for FLAG-GlyT1 or GlyT1-HA by using an anti-FLAG or anti-HA antibody, respectively. As shown in Supplemental Figure S1A,B, all GlyT1 forms observed in coimmunoprecipitation were found to be present in the cell lysates. Thus, we assumed that the anti-HA or anti-FLAG antibody favorably recognized GlyT1-HA or FLAG-GlyT1 dimers (a non-glycosylated form at ~140 kDa and a fully glycosylated form at ~190 kDa) and oligomers (more than 250 kDa) in anti-FLAG or anti-HA immunoprecipitates, but monomers (a fully glycosylated form at ~95 kDa and a non-glycosylated form at ~70 kDa) and a fully glycosylated dimer (~190 kDa) when the same antibody was used for both immunoprecipitation and immunoblotting.

### 2.2. Cross-Linking of GlyT1 L288C

**Figure 2:**
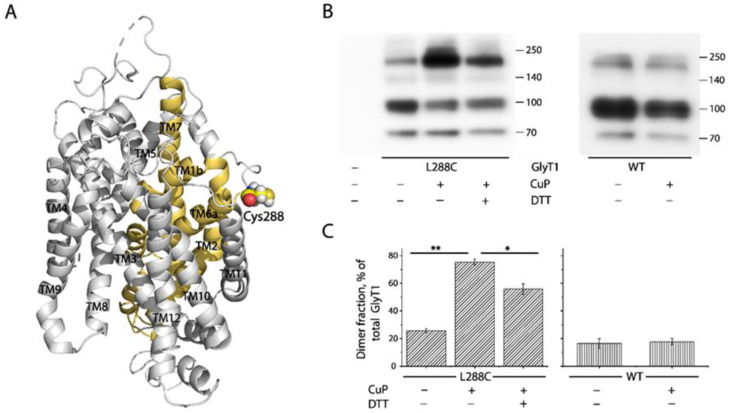
Location of the cysteine replacing Leu288 in GlyT1 and CuP cross-linking of the L288C mutant. (**A**) The cysteine replacing Leu288 in the x-ray structure of GlyT1 in an inward open conformation (PDB accession code, 6ZPL) [26]. Cys288 (spheres) is located at EL3 near the extracellular end of TM6 in the GlyT1 structure. The bundle domain (TMs 1, 2, 6, and 7) and other part of the protein are colored in golden and gray, respectively. (**B**) Immunoblots of WT (right panel) and L288C (left panel) with or without CuP treatment. HEK293 cells stably expressing WT or L288C were treated with 100 μM CuP at 22 °C for 15 min and the cross-linking reaction was then quenched by incubating them with 10 mM N-ethylmaleimide for 15 min. FLAG-tagged GlyT1 in total cell lysates was detected by immunoblot analysis as described under “Materials and Methods”. Numbers on the right of each blot represent molecular masses in kDa of protein standards. (**C**) Quantification of dimer fraction in total GlyT1. The fully glycosylated dimers (−190 kDa) were considered to reside on the cell surface, whereas non-glycosylated dimers were within the cytoplasm. Dimer fraction was expressed as percentage of fully glycosylated dimers in total DAT integrated density value. Results are shown as mean ± SEM averaged from three independent experiments. * *p* < 0.05; ** *p* < 0.01.

In hDAT, an endogenous cysteine residue, Cys306, located at the end of the third extracellular loop (EL3), connecting TM5 and TM6, is responsible for intermolecular disulfide bond formation upon oxidative treatment [38]. The corresponding residue in GlyT1b is Leu288 (Figure 2A). To investigate the effect of oxidation-induced dimerization on transport by GlyT1, we substituted Leu288 with cysteine. The *K_m_* for glycine and *K_d_* for CHIBA-3007 (a high-affinity GlyT1 selective ligand) in L288C were similar to those of WT GlyT1. *V_max_* and *B_max_* were about 65% and 62% of WT, respectively, largely due to a decreased surface expression of L288C compared to that of WT (Table 1).

To assess the intermolecular cross-linking of L288C, cells stably expressing L288C or WT were treated with an oxidative cross linker, copper phenanthroline (CuP) and lysed with RIPA buffer. The lysates were then subjected to immunoblot analysis for GlyT1 dimerization. As shown in Figure 2B, similar to WT, L288C without CuP treatment showed a small portion of fully glycosylated dimers (~190 kDa) in addition to fully glycosylated monomers (~95 kDa), immature GlyT1 (~70 kDa), and a trace of immature dimers (~140 kDa) when measured in total HEK293 cell lysates. CuP treatment did not alter WT GlyT1 distribution on a SDS-polyacrylamide gel (Figure 2B,C, blot and graph on right). By contrast, CuP treatment of L288C markedly increased the amounts of fully glycosylated dimers at ~190 kDa, accompanied by a significant decrease in the intensity of the fully glycosylated monomer band at ~95 kDa (Figure 2B, lane 3 and Figure 2C, 2nd column). Although DTT treatment did not completely eliminate CuP-induced disulfide bonds, dimer fraction of GlyT1 was significantly decreased (Figure 2B, lane 4 and Figure 2C, 3rd column), indicating that disulfide bond formation between L288C monomers accounted for CuP-induced dimerization.

CuP-induced oxidation has been reported only to cross-link cysteine pairs on the cell surface that are exposed to the extracellular medium [38]. Consistent with previous reports on hDAT [33,38], our results showed that CuP cross-linking of GlyT1 L288C only produced dimerization of mature L288C (~95 kDa) but not its immature form (~70 KDa), suggesting that CuP cross-linking of GlyT1 occurred on the cell surface.

### 2.3. Functional Consequences of CuP Cross-Linking of L288C

**Figure 3:**
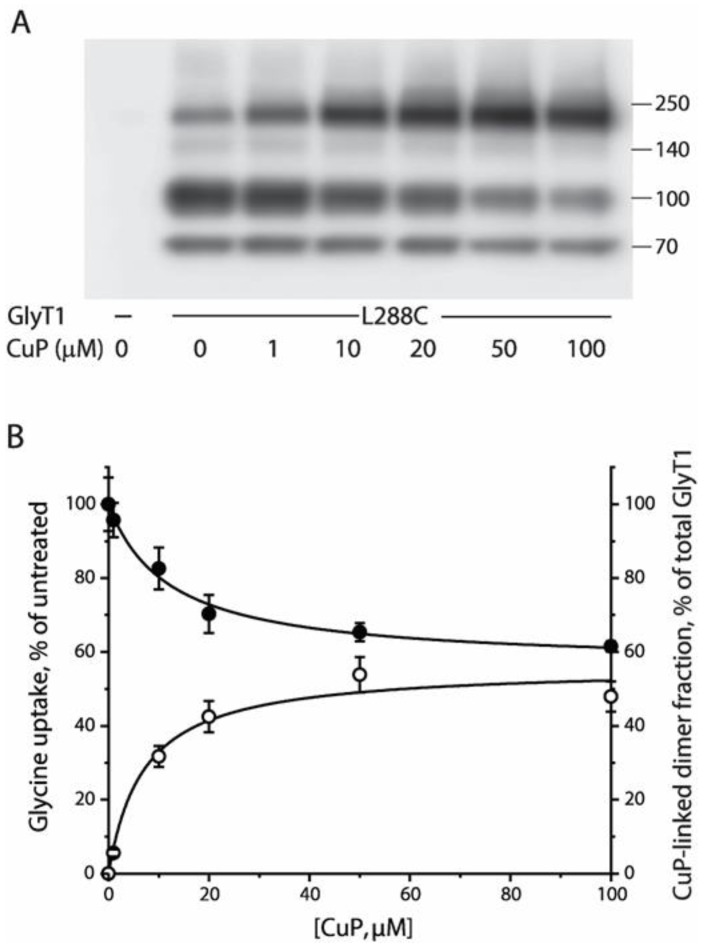
CuP concentration-dependent cross-linking and its influence on transport activity of GlyT1 L288C. (**A**) The representative immunoblot of CuP cross-linking of L288C. HEK-L288C cells were treated without or with CuP at indicated concentrations at 22 °C for 15 min and then lysed. The FLAG-tagged L288C in cell lysates were detected by immunoblot analysis using anti-FLAG antibody. (**B**) Relationship between CuP-induced dimer fraction of GlyT1 (◦) or glycine uptake (●) and CuP concentrations. After HEK-L288C cells were treated with varying concentrations of CuP, [^3^H]glycine uptake measurements and immunoblot analysis were parallelly performed. [^3^H]Glycine uptake was expressed as a percentage of the value obtained without CuP treatment and CuP-induced dimer fraction was expressed as an increased dimer percentage caused by CuP cross-linking after subtracting a spontaneously occurring dimer percentage of total GlyT1 integrated density value. All results are presented as mean ± SEM averaged from three independent experiments.

To assess the effect of CuP cross-linking on L288C transport function, we examined, in parallel, CuP-induced dimerization and its influence on transport activity. As shown in Figure 3A, the intensity of the ~190 kDa band gradually increased in a CuP concentration-dependent manner. This was accompanied by a decrease in the band intensity of the fully glycosylated GlyT1 monomer (~95 kDa). CuP-induced dimer fractions, expressed as the increase, in percent, of fully glycosylated dimers found in total cell lysates (% dimer fraction), were plotted as a function of CuP concentrations (Figure 3B, open circles). The maximally increased % dimer fraction we observed over a range of CuP concentrations (1 to 100 μM) was 55.9 ± 3.6%, and the CuP concentration leading to half-maximal dimerization was 7.0 ± 2.1 μM. In parallel, we measured the transport rate after CuP treatment at the same concentrations (Figure 3B, filled circles). The results indicated a progressive loss of glycine uptake as more L288C dimers were formed by CuP cross-linking. The transport activity remaining at the highest CuP concentration was estimated to be 54.0 ± 2.8% of control measurements in the absence of CuP. The CuP concentration leading to half-maximal inactivation was 12.1 ± 2.4 μM, similar to the concentration resulting in half-maximal dimerization. The CuP-induced dimer, thus, was considered to be inactive to transport glycine.

We then performed kinetic analysis to characterize the effects of CuP cross-linking of L288C on glycine transport and CHIBA-3007 binding. Our results showed that *V_max_* for glycine uptake was significantly decreased with little change in *K_m_* by 100 μM CuP treatment (Table 1), suggesting that the spontaneously occurring dimers of L288C acted normally. Unlike glycine uptake, the *B_max_* for [^3^H]CHIBA-3007 binding was not changed after CuP treatment, indicating the ligand can bind to both the CuP-induced and spontaneously occurring L288C dimers. However, the *K_d_* value was increased with CuP cross-linking by 2.8 folds, suggesting that CuP-induced L288C dimers have a lower affinity for CHIBA-3007 than spontaneously occurring L288C dimers (Table 1).

### 2.4. Influence of Intramolecular K286-E289 Ion Pair on CuP Cross-Linking of L288C

**Figure 4:**
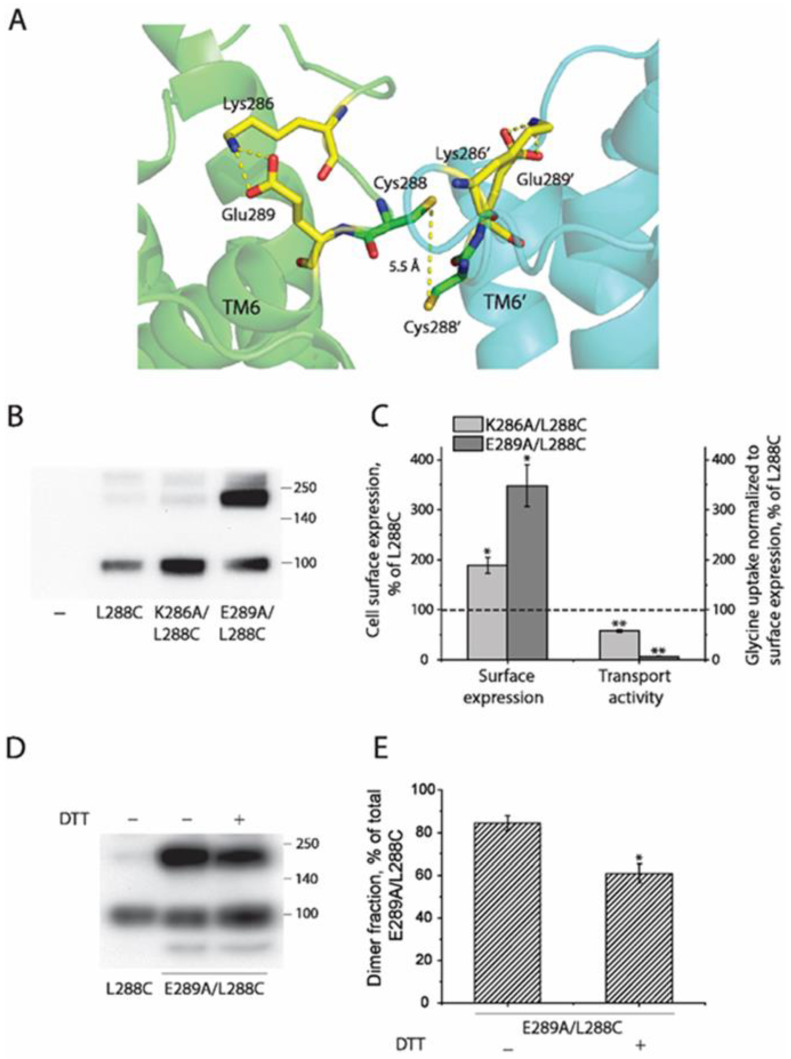
The intramolecular salt bridge in EL3 modulates dimeric interfacial interactions and transport activity of GlyT1 L288C. (**A**) Dimer docking of GlyT1 L288C with each protomer in an inward open conformation (PDB accession code, 6ZPL). An intramolecular salt bridge formed by Lys286-Glu289 in EL3 near the extracellular end of TM6 was shown in each protomer. The two protomers in a putative GlyT1 L288C dimer are colored in green and cyan, respectively. (**B**) A representative immunoblot of biotinylated L288C, K286A/L288C, and E289A/L288C. HEK293 cells stably expressing GlyT1 L288C, K286A/L288C, or E289A/L288C were treated with sulfo-NHS-SS-biotin at 4 °C for 20 min. After precipitation with streptavidin agarose, biotinylated GlyT1 was detected by immunoblot analysis as described under “Materials and Methods”. (**C**) Quantification of cell surface expression and transport activity of GlyT L288C, K286A/L288C, and E289A/L288C. Biotinylation and [^3^H]glycine uptake assays were performed in parallel to measure cell surface expression and transport activity of GlyT1 L288C, K286A/L288C, and E289A/L288C. Cell surface expression was expressed as a percentage of the integrated density value of GlyT1 L288C. Transport activity was expressed as a percentage of [^3^H]glycine uptake normalized to cell surface expression of GlyT1 L288C. The dotted line shows cell surface expression and transport activity of GlyT1 L288C. All results are presented as mean ± SEM averaged from three independent experiments. * *p* < 0.05; ** *p* < 0.01, compared to L288C. (**D**) A representative immunoblot of the E289A/L288C mutant with or without DTT treatment. HEK293 cells stably expressing E289A/L288C were lysed, and the cell lysates were then treated with or without 100 mM DTT at 22 °C for 1 h. GlyT1 E289A/L288C was detected by immunoblot analysis. (**E**) Quantification of dimer fraction of E289A/L288C with or without DTT treatment. Dimer fraction was expressed as a percentage of fully glycosylated dimers relative to total GlyT1 E289A/L288C. Results are presented as mean ± SEM averaged from three independent experiments. * *p* < 0.05 compared to E289A/L288C without DTT treatment.

We conducted protein–protein docking to generate a L288C dimeric model using the GlyT1 crystal structure, which has been recently resolved as a monomer bound with a benzylpiperazine chemotype inhibitor in an inward open conformation [26]. In our dimeric model, Cys288 cysteines between two protomers are in close proximity, only 5.5 Å apart, allowing the possibility of disulfide bond formation (Figure 4A). It is notable that there is an intramolecular ion pair (K286-E289) to form a salt bridge that is expected to stabilize the EL3 in a conformation that keeps the two Cys288 residues at a cross-linking distance. The ion pair residues are highly conserved through the NSS family [40,41,42]. To test the importance of the salt-bridge in GlyT1 L288C dimerization, we substituted each of the ion pair residues with alanine, one at a time, and investigated the effects of these substitutions on oxidation-induced L288C dimerization and transport function.

Compared with the background construct (L288C), replacement of Lys286 or Glu289 with alanine dramatically increased its cell surface expression by approximately 1.9- or 3.5-fold, respectively (Figure 4B,C). Strikingly, immunoblot analysis after surface biotinylation showed that E289A/L288C formed predominant dimers on the cell surface (Figure 4B), which can be partially cleaved by DTT treatment (Figure 4D,E). The two mutants, K286A/L288C and E289A/L288C, however, showed a markedly decreased transport activity. As shown in Figure 4C, transport activity of K286A/L288C or E289A/L288C normalized to its surface expression was 58% or 7% of the L288C background, respectively. These results suggested that disruption of the K286-E289 salt bridge by these mutations increased the interface interactions between the two L288C protomers, resulting in an increased stability in the cell membrane, decreasing internalization and subsequent degradation while also causing a spontaneous Cys288-Cys288’ disulfide bond formation between the two E289A/L288C protomers. Moreover, an increase in the interface interactions in a dimer of either K286A/L288C or E289A/L288C could possibly account for impairment of their transport function.

**Figure 5:**
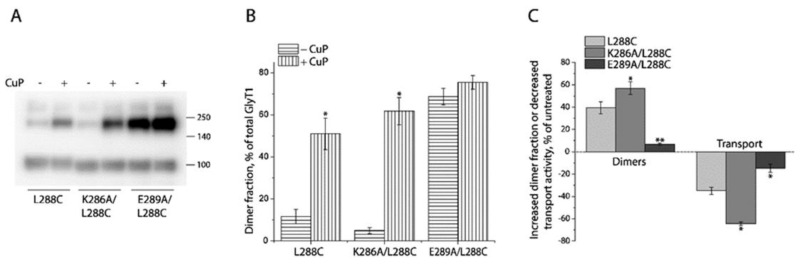
Influence of ion pair mutations on CuP cross-linking and transport activity of L288C. (**A**) A representative immunoblot of ion pair mutants with or without CuP treatment. HEK293 cells stably expressing GlyT1 L288C, K286A/L288C, or E289A/L288C were treated with 10 μM CuP at 22 °C for 15 min. After quenching, GlyT1 in cell lysates was detected by immunoblot analysis as described under “Materials and Methods”. (**B**) Quantification of dimer fractions of L288C, K286A/L288C, and E289A/L288C with or without CuP treatment. Dimer fraction was expressed as a percentage of fully glycosylated dimers in total GlyT1. Results are presented as mean ± SEM averaged from three independent experiments. * *p* < 0.05 compared to each mutant without CuP treatment. (**C**) CuP-induced increase in dimer fraction and decrease in transport activity. Immunoblot analysis and [^3^H]glycine uptake were parallelly performed to examine CuP-induced changes in both dimer fraction and transport activity with HEK293 cells stably expressing GlyT1 L288C, K286A/L288C, and E289A/L288C as described under “Materials and Methods”. All results are presented as mean ± SEM averaged from at least three independent experiments. * *p* < 0.05; ** *p* < 0.01 compared to the background construct, L288C.

Then, we investigated the effects of mutating the ion pair residues on CuP-induced cross-linking and transport activity. CuP treatment significantly increased oxidation-induced dimers in K286A/L288C (Figure 5A,B), and the increased dimer fraction in K286A/L288C was statistically greater than that observed with its parental construct L288C (Figure 5C). By contrast, CuP-induced cross-linking in E289A/L288C was much less than that in either the background construct or K286A/L288C (Figure 5A–C), due to a prominent spontaneous cross-linking. In addition, the increased dimer fraction by CuP cross-linking in these mutants led to proportional reduction of their transport activity (Figure 5C).

### 2.5. Influence of Zn^2+^ on CuP Cross-Linking of L288C

**Figure 6:**
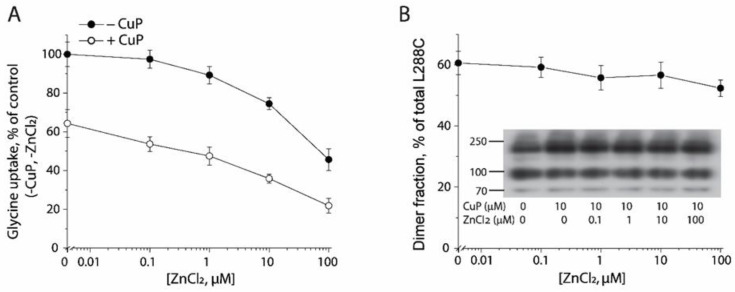
Influence of Zn^2+^ on CuP cross-linking and transport activity of L288C. (**A**) Influence of CuP on Zn^2+^ induced decrease in transport activity of L288C. HEK293 cells stably expressing L288C were treated with ZnCl_2_ at indicated concentrations in the absence or presence of 10 μM CuP at 22 °C for 15 min and [^3^H]glycine uptake was measured as described under “Materials and Methods”. The graph showed a representative experiment with triplicate measurements. The experiment was repeated twice with similar results. Glycine uptake was expressed as a percentage of glycine uptake of L288C without both CuP and ZnCl_2_ treatments. (**B**) Influence of ZnCl_2_ on CuP-induced cross-linking of L288C. HEK293 cells stably expressing L288C were treated with ZnCl_2_ at indicated concentrations in the absence or presence of 10 μM CuP at 22 °C for 15 min. After quenching, L288C in cell lysates was detected by immunoblot analysis. A representative immunoblot was shown (inset). Dimer fraction was expressed as a percentage of fully glycosylated dimers in total L288C. All results are presented as mean ± SEM averaged from three independent experiments.

An earlier report demonstrated that Zn^2+^ binding to an endogenous Zn^2+^ binding site in GlyT1, composed of His243 in EL2 and His410 in EL4, noncompetitively inhibited GlyT1 through its influence on substrate translocation [43]. Similar inhibitory modulation of transport function by Zn^2+^ binding was also observed with DAT [44,45]. Furthermore, Zn^2+^ binding to an engineered site at a predicted dimeric interface of DAT involving an inserted His310 and an endogenous Cys306 in the extracellular end of TM6, potently inhibited DAT transport activity [46]. In light of the strong inhibitory effect on transport function by Zn^2+^ acting in the dimeric interface of DAT, we asked if Zn^2+^ influences CuP cross-linking of GlyT1 L288C. In agreement with earlier reports for GlyT1 and DAT [43,44,45,46], increasing concentrations of Zn^2+^ gradually decreased glycine uptake (Figure 6A, filled circles). A similar curve for Zn^2+^ inhibition was also observed after treatment of 10 μM CuP (Figure 6B, open circles), although overall transport activity was lower. These results suggest that Zn^2+^ had little effect on CuP-induced inhibition of L288C transport activity. The results are also consistent with the finding that CuP-induced dimers of L288C were not altered by Zn^2+^ addition in the immunoblot analysis (Figure 6B).

### 2.6. Influence of GlyT1 Conformation on CuP Cross-Linking of L288C

**Figure 7:**
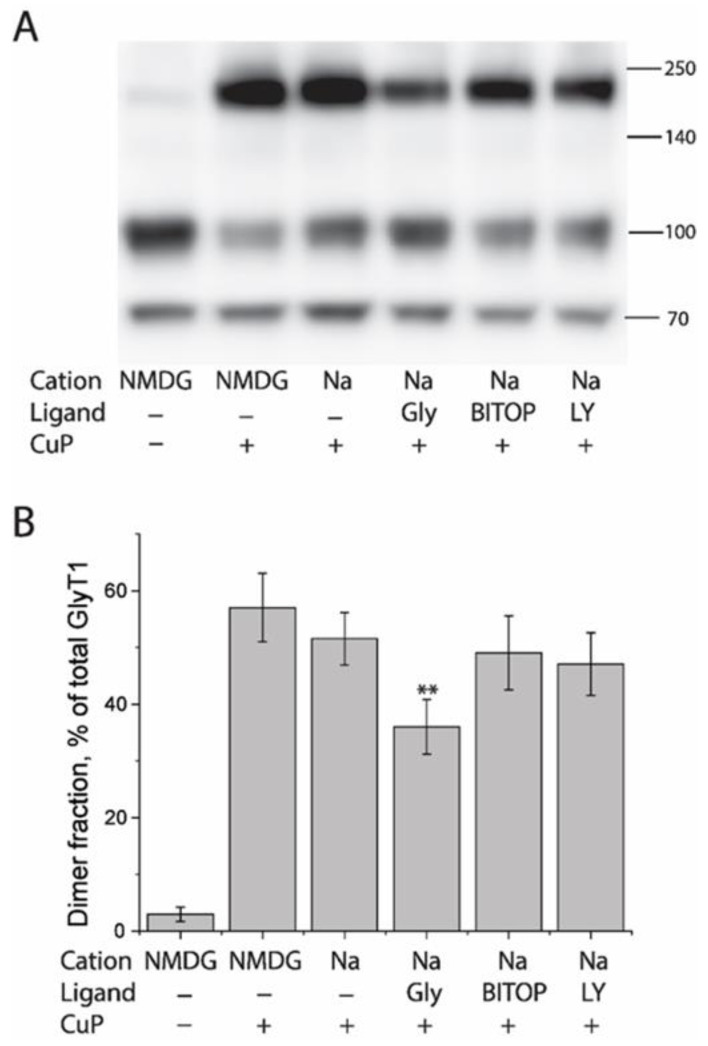
Influence of GlyT1 conformations on CuP cross-linking of L288C. (**A**) A representative immunoblot showing CuP cross-linking of L288C under different conditions. HEK293 cells stably expressing L288C were treated with 10 μM CuP in the absence or presence of the indicated cation (150 mM), glycine (200 μM), or ligand (10 μM) at 22 °C for 15 min. After quenching, L288C in total lysates was detected by immunoblot analysis. (**B**) Quantification of dimer fraction under indicated treatments. The fraction of dimers was expressed as a percentage of fully glycosylated dimers. All results are presented as mean ± SEM averaged from three independent experiments. ** *p* < 0.01 compared to the value obtained with L288C under CuP treatment in the presence of 150 mM NMDGCl. Gly, glycine; BITOP, bitopertin; LY, LY2365109.

The CuP-induced L288C dimer was not able to transport glycine, presumably due to the spatial restriction of Cys288 movement resulting from the formation of an intermolecular disulfide bond. This result raises a possibility that Cys288 is conformationally sensitive to CuP cross-linking during the transport cycle. To test this possibility, we investigated the effect of transporter conformations on CuP cross-linking of L288C. In this assay, we used Na^+^ and a competitive inhibitor LY2365109 [47,48], a non-competitive inhibitor, bitopertin [49,50], and the substrate, glycine, to induce GlyT1 in various conformations. As shown in Figure 7, in comparison with the control (CuP treatment in the presence of NMDGCl), neither Na^+^ nor GlyT1 inhibitors influenced CuP cross-linking of L288C. On the other hand, addition of glycine in the presence of NaCl led to a significant decrease in CuP-induced dimer fraction (Figure 7A, 4th lane and Figure 7B, 4th column), suggesting that the effect of substrate transport on GlyT1 conformation reduced the likelihood of Cys288 residues at the dimer interface to be within cross-linking distance.

## 3. Discussion

In the NSS family, SERT, DAT, NET, and GAT have been demonstrated to form dimers and even higher order oligomers by using various biochemical approaches [35]. Earlier reports on GlyT1 and GlyT2, however, diverged from this rule, as both isoforms were indicated to exist only as monomers at the plasma membrane based on hydrodynamic and native gel electrophoretic studies [51]. Subsequently, another study used a combination of cross-linking and fluorescence resonance energy transfer analysis in living cells, to demonstrate that these transporters are dimers not only in the intracellular compartments but also at the plasma membrane of HEK293T cells [39]. The results presented here show that detergent-solubilized GlyT1s associate each other in a coimmunoprecipitation assay, in which two GlyT1 constructs, each with a different epitope tag, were coimmunoprecipitated (Figure 1). Immunoblots of these precipitates show prominent bands consistent with the mobility of GlyT1 dimers and higher order oligomers. Additionally, oxidation with CuP enhances the intensity of the dimer band in a mutant with a sensitive cysteine in a proposed dimer interface (Figure 2). It is not known if GlyT1 dimerization or oligomerization involves disulfide cross-linking in vivo. However, the fact that these disulfides readily form when a cysteine replaces Leu288, combined with the observation of dimers or oligomers in the absence of a cysteine at that position, suggests that GlyT1 dimerization or oligomerization may involve at least the face of the protein containing on which Leu288 resides. The evidence presented here supports the proposal that GlyT1 forms dimers and higher order oligomers, in which Leu288 contributes to the dimeric or oligomeric interface.

Despite efforts to identify GlyT dimers, experimental evidence for the influence of dimerization or oligomerization on transport activity is sparse [39]. In this study, we measured a parallel dimerization and functional inhibition of GlyT1 L288C induced by CuP (Figure 3). The relationship between transport activity and dimerization on the cell surface (Table 1) indicated that the increase in CuP-induced dimerization of L288C is proportional to its reduction in transport function. This finding is not unique. Several members of the NSS family, such as, DAT, NET, and SERT have been shown to lose transport activity with dimerization [27,28,32].

Formation of a DAT dimer has been reported to decrease transport activity more than reported here for the GlyT1 L288C mutant [32]. Cross-linking of Cys288 into dimers in GlyT1 resulted in a maximal inactivation of transport activity by 46% (Figure 3), whereas cross-linking of the corresponding residue, Cys306, in DAT inactivated up to 96% of transport activity. The difference in inhibition of functional activity between GlyT1 and DAT dimers could result from different dimeric interface interactions. There are also other differences between the two transporters in the effect of dimerization on functional properties. Disruption of the proposed salt bridge by substituting the ion pair residues with alanine (R304A and E307A) led to a significant increase in specific transport activity and a reduction in CuP-induced disulfate bond formation at Cys306 in DAT [40]. The present work, however, indicates that the same substitutions at the corresponding positions (K286A and E289A) markedly increased their dimerization by cross-linking of Cys288 in GlyT1 and impaired their transport activity (Figure 5). Despite these opposite effects, the studies agree in that nearby electrostatic interactions are important for stabilizing the transporter proteins in a conformation that allows cross-linking and favors dimerization. Furthermore, protein docking and molecular dynamics simulation suggest that Arg304 and Glu307 in a DAT dimer appear to form intermolecular salt bridges that potentially make additional interface contacts [42]. By comparison, the corresponding ion pair residues Lys286-Glu289 in GlyT1 favor to form an intramolecular salt bridge (Figure 4). Thus, the difference in the ion pair interactions between DAT and GlyT1 might be a key reason for different impacts of dimerization on their function.

Leu288 in GlyT1 is located at EL3 near the extracellular end of TM6, a part of bundle domain that participates in substrate and ion binding (Figure 2). Comparison of the coordination of substrate and ion binding sites in the monomeric structure with that in the dimeric model of GlyT1, shows that the dimeric interface does not have a direct interaction with the substrate and ion binding sites, consistent with our observation that CuP cross-linking of L288C decreased the *V_max_* for glycine transport but with little change in the *K_m_* for glycine (Table 1). Similar results were also seen with other transporters in the NSS family [27,28,32], supporting the proposal that CuP-induced dimers at the equivalent position are inactive to transport substrates with little change in substrate binding affinity.

**Figure 8:**
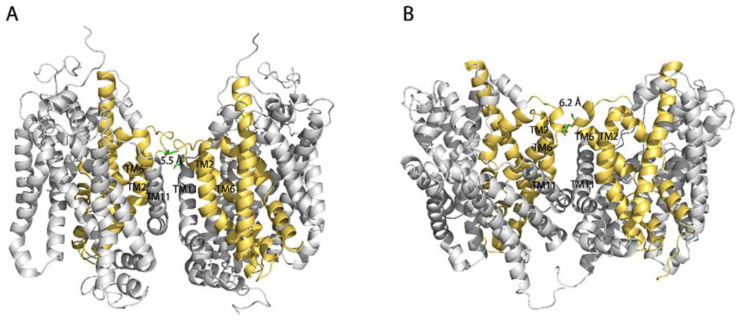
Dimer docking of GlyT1 L288C with both protomers in an inward open (**A**) or an outward open conformation (**B**). Dimer docking was performed based on the crystal structure of GlyT1 in an inward open conformation (PDB accession code, 6ZPL) [26] and the GlyT1b model in an outward open conformation. The distance between two dimeric cysteines replacing Leu288 in the inward-open dimer and outward-open dimer are 5.5 and 6.2 Å, respectively. The bundle domains (TMs 1, 2, 6, and 7) and other part of the protein are colored in golden and gray, respectively.

Our protein docking based on the monomeric structure in an inward open conformation [26] or a monomeric model in an outward open conformation indicated that the distance between the two Cys288 residues in a GlyT1 L288C dimer was 5.5 Å or 6.2 Å, respectively, in favor of a possible disulfide bond formation (Figure 8). The dimeric models of GlyT1 L288C also showed that interfacial Cys288 contacts between two protomers are complemented by an association between TM2, TM6, and TM11 portions (Figure 8). In addition, comparison of the dimeric models revealed that the protomers are clearly tilted away from each other in an outward open-dimer model, but the distance between the two Cys288 residues are little changed. Consistent with these computations, ligands or ions that stabilize GlyT1 in either an outward open conformation, such as Na^+^ [52] or LY2365109 [53], or an inward open conformation, such as bitopertin [26], had little effect on CuP cross-linking of Cys288 residues. By contrast, the substrate, glycine, in the presence of both Na^+^ and Cl^−^, significantly reduced the oxidative cross-linking of L288C (Figure 7), indicating the rapid conformational conversions caused by a large-scale repeated rotation of the bundle domain during substrate transport impair the dimeric interface interactions, and thus diminish transient contacts between the two Cys288 residues in a GlyT1 L288C dimer. Our biochemical results support the reciprocal correlation between dimerization and transport function.

It has been demonstrated that oxidative cross-linking of DAT can also occur between Cys243 residues in TM4, which is positioned on the opposite site on the transporter from Cys306 [54]. In addition, a comprehensive molecular dynamics simulation revealed that DAT probably forms eight dimer configurations, which are clustered into four symmetric and four asymmetric geometries [42]. Two symmetric dimers in which Cys306 and Cys243 serve as the cross-linked residues in the dimeric interface, respectively, have been biochemically validated [38,42]. These studies raise the possibility that DAT forms multiple conformational clusters of dimers. For GlyT1, it is also of interest to learn if GlyT1 can form dimers with multiple configurations and how different dimerization influences transport function, which would provide structural elements for understanding of higher order oligomerization and its functional consequences.

## 4. Materials and Methods

### 4.1. Materials

HEK293 cells were from American Type Culture Collection. N-terminal FLAG-tagged human GlyT1b (FLAG-GlyT1b) expression plasmid was constructed in pcDNA3.1 under control of the CMV promotor, as described previously [52]. A C-terminal HA-tagged GlyT1b (GlyT1b-HA) was also constructed in pcDNA3.1 in this study. Lenti-EF-1α-BSD vector with a blasticidin resistance and two packaging vectors (psPAX2 and pMD2G) were generous gifts from Dr. G. Wang (Guangzhou University). Anti-FLAG M2 agarose gel, anti-HA agarose gel, monoclonal antibodies for anti-FLAG and anti-HA, 3 × FLAG peptide, and HA peptide were from Sigma-Aldrich (St. Louis, MO, USA). [^3^H]Glycine (42 Ci/mmol) was purchased from PerkinElmer (Boston, MA, USA). [^3^H]CHIBA-3007 (67 Ci/mmol) was a product synthesized previously [52]. LipoJet was obtained from Signagen (Frederick, MD, USA). Sulfosuccinimidyl 2-(biotinamido)ethyl-1,3-dithiopropionate (sulfo-NHS-SS-biotin), streptavidin agarose gel, and Super Signal West Pico were purchased from ThermoFisher Scientific (Waltham, MA, USA). All other reagents were of analytical grade.

### 4.2. Mutagenesis

The full-length cDNA of GlyT1b was amplified using FLAG-GlyT1b in pcDNA3.1 as a template by PCR and inserted into the Xba I and BamH I sites of lenti-EF-1α-BSD vector by Exnase II. GlyT1b mutants used for cross-linking study in this work were constructed in the N-terminal FLAG-tagged WT GlyT1b background carried by the lentiviral plasmid, lenti-EF-1α-GlyT1b-BSD. All mutants were generated using the Mut Express II Fast Mutagenesis Kit (Vazyme) and confirmed by full-length DNA sequencing.

### 4.3. Lentivirus and Stable Cell Line Preparation

The lentivirus for WT GlyT1b and mutants were prepared using HEK293T cells, respectively, as described previously [55]. In brief, HEK293T cells at 70–80% confluence were transfected by a mixture of the lentiviral plasmid and other two packaging vectors, psPAX2 and pMD2G, using LipoJet transfection reagent. Virus was collected into a 10 mL tube 48 h after transfection, followed by centrifugation to remove cell debris at 500 × g for 10 min. The supernatant was then filtered through a 0.45 μm filter and stored at −80 °C until further use.

HEK293T cells were infected by the lentivirus with polybrene in the complete Dulbecco’s Modified Eagle’s Medium (DMEM) with blasticidin S at a concentration of 12 μg/mL. The medium was replaced every 3 days until colonies of blasticidin S-resistant cells were formed. The cells were maintained in DMEM supplemented with 10% fetal bovine serum, 100 units/mL penicillin, 100 μg/mL streptomycin, and 12 μg/mL blasticidin S at 37 °C in a humidified 5% CO_2_ incubator and were then plated in 12-well culture plates. The stable cell lines expressing GlyT1b were confirmed by immunoblot analysis for the transporter expression. Protein concentration was determined with the Micro BCA protein assay reagent kit.

### 4.4. Transport and CHIBA-3007 Binding Assays

[^3^H]Glycine transport was measured in 96- or 24-well plates using HEPES buffered saline (10 mM HEPES, 1.2 mM MgSO_4_, 2 mM K_2_SO_4_, 10 mM glucose, and 150 mM NaCl, pH 7.4) as described previously [56]. The extent of [^3^H]glycine accumulation was determined with a PerkinElmer MicroBeta plate counter or Beckman LS6500 liquid scintillation counter. Binding of [^3^H]CHIBA-3007 was measured in cell membranes prepared from the cells expressing GlyT1b or its mutants in 96-well filter plates using binding buffer (10 mM HEPES buffer, pH 7.4, containing 150 mM NaCl), according to the protocol as described previously [57]. CHIBA-3007 binding was measured using a PerkinElmer MicroBeta counter after incubation with [^3^H]CHIBA-3007 at a final concentration of 1 nM for 1.5 h at room temperature.

### 4.5. Co-Immunoprecipitation and Immunoblotting

The cells co-expressing FLAG-GlyT1b and GlyT1b-HA in a 6-well plate were lysed with 1 mL of RIPA buffer containing 50 mM Tris, 5 mM EDTA, 150 mM NaCl, 1% Triton X-100, pH 7.5, and the detergent extracts were incubated with 25 μL of anti-FLAG M2 or anti-HA agarose gel at 4 °C overnight. The immunoadsorbents were washed three times with ice-cold RIPA buffer before elution with 100 μL of elution buffer (150 ng/μL 3 × FLAG peptide or 100 ng/μL HA peptide in RIPA buffer). Protein samples were separated by a 10% SDS-polyacrylamide gel, transferred to a PVDF membrane (Bio-Rad, Hercules, CA, USA), and probed with specific antibodies (1:1000) as indicated. A horseradish peroxidase-conjugated anti-mouse IgG (1:10,000) was used to visualize the signal by Super Signal West Pico with a UVP Biochemi II imaging system. The blots were then stripped by stripping buffer (ThermoFisher Scientific, Waltham, MA, USA), and reprobed with indicated antibodies. Immunoreactive bands were visualized by chemiluminescence.

### 4.6. Biotinylation

Surface expression of WT GlyT1b and its mutants was determined using the membrane-impermeant biotinylation reagent sulfo-NHS-SS-biotin as described previously [58]. HEK293T cells stably expressing GlyT1b or its mutants were treated twice with sulfo-NHS-SS-biotin for 20 min on ice. After labeling, the cells were rinsed with 100 mM glycine in phosphate-buffered saline (PBS) containing 137 mM NaCl, 2.7 mM K_2_SO_4_, 4.3 mM Na_2_HPO_4_, and 1.4 mM KH_2_PO_4_, pH 7.4 for 20 min on ice to quench excess sulfo-NHS-SS-biotin. The cells were then lysed, and the biotinylated proteins were recovered using streptavidin-agarose beads in an overnight incubation at 4 °C with gentle agitation. The beads were washed, and the biotinylated proteins were eluted with 100 μL of SDS-PAGE sample buffer. Samples were applied to a 10% SDS-polyacrylamide gel and visualized by immunoblotting. The transporters were detected using anti-FLAG monoclonal M2 antibody (1:1000) against the FLAG epitope tag at the N-terminus of GlyT1b. Immunoreactive bands were visualized by chemiluminescence, and the amount of surface expression was quantified using a UVP Biochemi II imaging system.

### 4.7. Oxidative Cross-Linking of GlyT1b

HEK293 cells stably expressing GlyT1b or its mutants in a 6-well plate were washed twice with HEPES buffered saline and incubated with copper phenanthroline (CuP, a mixture of CuSO_4_ and 1,10- phenanthroline) at indicated concentrations for 15 min at 22 °C, as described previously [59,60]. The cross-linking reaction was stopped by 2 × rapid washing with HEPES buffered saline and incubating with 10 mM N-ethylmaleimide for 15 min, followed by three more washes. The cells were lysed and the lysates in a non-reducing sample buffer were then assayed for dimerization of GlyT1b by immunoblot analysis. To investigate DTT cleavage of CuP-induced disulfate bond formation, cell lysates were incubated with 100 mM DTT at 22 °C for 1 h prior to immunoblot analysis.

To measure the effect of ions or ligands on CuP cross-linking, ligands were added to the cells and preincubated for 5 min in HEPES binding buffer containing 150 mM N-methyl-D-glucamine (NMDG) chloride, sodium isethionate, or NaCl as indicated. CuP cross-linking was performed in the presence of indicated ions or ligands.

### 4.8. Homology Modeling

A homology model of GlyT1b in an outward open conformation was generated with Modeller [61] based on an outward-facing structure (2.89 Å resolution, PDB accession code, 4XP1) of the *Drosophila melanogaster* DAT [21]. The alignment between two sequences was obtained with ClustalW and covered residues 31 to 168 and 188–581 for hGlyT1b, that is, all except a portion of extracellular loop 2 and the N and C termini. The sequence identity between hGlyT1b and dDAT is 45.71%. The models with the highest scores were selected for visualization. Figures of structure and model were generated using PyMOL v2.5.2 (Schrödinger, Inc.).

### 4.9. Dimer Docking of GlyT1b

The X-ray crystal structure of GlyT1 bound with a noncompetitive inhibitor in an inward open conformation (PDB accession code, 6ZPL) [26] and GlyT1b model in an outward open conformation were used for generating dimers using the dimer docking module in ClusPro 2.0 [62,63,64,65,66], respectively. The residues were numbered according to the positions in hGlyT1b. We performed dimer docking by using Leu288 as an anchoring point, which generated more than 30 dimer models. We selected one pose with the best score and exported it into PyMOL for visualization.

### 4.10. Data Analysis

Nonlinear regression fits of experimental and calculated data were performed with Origin (Origin Lab). The statistical analysis given was from multiple experiments. Data with error bars in the figures represent the mean ± SD for triplicate measurements per condition in one experiment or mean ± SEM for at least three experiments as indicated, respectively. Statistical analysis was performed using Student’s paired *t*-tests.

## 5. Conclusions

The present study investigated oxidative cross-linking of a GlyT1 cysteine mutant L288C into dimers and its influence on transport function by using biochemical and structural approaches. Our results indicated that CuP-induced dimerization of L288C inhibits its transport activity. In addition, the molecular factors influencing L288C dimerization and consequently regulating its transport function were examined. An intramolecular ion pair, K286-E289, was demonstrated to play a key structural role in regulating the dimeric interface interactions and transport activity. Furthermore, the large-scale structural rearrangements of the bundle domain in response to substrate transport was proposed to lead to the impairment of dimeric interface interactions in the GlyT1 L288C mutant. These results are expected to provide insights into structural and functional elements for our understanding of the regulatory mechanism of GlyT1 transport activity through its dimerization or oligomerization.

## Figures and Tables

**Table 1:** Kinetic parameters of [^3^H]glycine uptake and [^3^H]CHIBA-3007 binding with or without CuP treatment.

	*K_m_* (μM)	*V_max_* (pmol/mg/min)	Cell Surface Expression (% of WT)	*K_d_* (nM)	*B_max_* (pmol/mg)
WT	38.6 ± 2.6	1413 ± 40	100	5.70 ± 0.47	2.76 ± 0.09
L288C	43.6 ± 2.5	918.5 ± 20 *	68.3 ± 5.3 *	7.38 ± 0.43	1.73 ± 0.07 *
L288C + CuP	54.1 ± 1.8	598.8 ± 17 ^#^	nd	20.6 ± 1.4 ^#^	1.88 ± 0.07

* *p* < 0.05 compared to WT; ^#^ *p* < 0.05 compared to L288C without CuP treatment. [^3^H]glycine uptake was measured over a range of glycine concentrations (50 nM–200 μM) with cells expressing WT or L288C as described under “Materials and Methods”. [^3^H]CHIBA-3007 binding measurements were performed over a range of CHIBA-3007 concentrations (1–100 nM) using membrane preparations from the cells expressing WT or L288C as described under “Materials and Methods”. For CuP treatment, CuP at a final concentration of 100 μM was added to the cells for 15 min at 22 °C and then washed away. Results are shown as mean ± SEM averaged from three independent experiments in triplicates; nd, not determined.

## Data Availability

The data presented in this study are available on request from the corresponding author.

## Supplementary Materials

The following supporting information can be downloaded at https://www.mdpi.com/article/10.3390/ijms232416157/s1, Figure S1: Immunoblot analysis for FLAG-GlyT1 and GlyT1-HA in total cell lysates.
