# Supplementary material for: Cross-Linking and Functional Analyses for Dimerization of a Cysteine Mutant of Glycine Transporter 1
Source: Int J Mol Sci. 2022 Dec 18;23(24):16157. doi: 10.3390/ijms232416157 (PMC9781295; doi:10.3390/ijms232416157)
Supplement: Supplementary file 1 [file ijms-23-16157-s001.zip › ijms-2047985-supplementary.pdf]

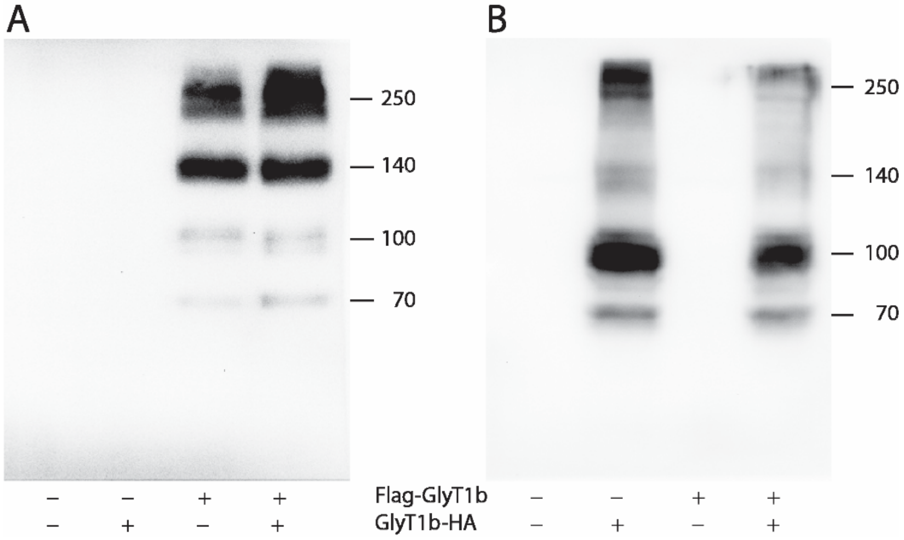

Figure S1. Immunoblot analysis for FLAG-GlyT1 and GlyT1-HA in total cell lysates. The total lysates from the cells expressing FLAG-GlyT1, GlyT1-HA, or both were separated by a SDS-polyacrylamide gel, transferred to a PVDF membrane, and probed with anti-FLAG M2 (**A**) or anti-HA antibody (B). Immunoreactive FLAG-GlyT1 or GlyT1-HA bands were visualized by chemiluminescence.
